# Alexithymia, reward sensitivity and excessive exercise in non-binge-eaters versus severe binge eaters: Implications for primary and secondary exercise dependence

**DOI:** 10.1007/s12144-022-03511-2

**Published:** 2022-08-10

**Authors:** Michael Lyvers, Joseph Truncali, Peta Stapleton, Fred Arne Thorberg

**Affiliations:** Bond University, Gold Coast, Qld 4229 Australia

**Keywords:** Exercise dependence, Alexithymia, Reward sensitivity, Interoception, Mood

## Abstract

A distinction has been made between primary and secondary exercise dependence, with the latter defined as excessive exercise secondary to disordered eating and weight concerns. Based on theoretical considerations from research on the roles of trait factors in addictions, the present study used validated scales to assess alexithymia, sensitivity to reward and punishment, emotion regulation and interoception in relation to exercise dependence symptoms in Australian male and female non-binge eaters (*n* = 228) and severe binge eaters (*n* = 126) aged 18–30 yr. In both groups, exercise dependence symptoms were significantly positively associated with reward sensitivity and interoceptive awareness, with the latter two variables predicting exercise dependence symptoms in hierarchical regression models; punishment sensitivity was significantly negatively related to such symptoms. Alexithymia was significantly associated with exercise dependence symptoms only in non-binge eaters; in severe binge eaters, alexithymia explained 0% of unique variance. Male sex was associated with more exercise dependence symptoms in severe binge eaters only. Participants in the severe binge group scored significantly higher on measures of exercise dependence, alexithymia, risky alcohol use, and sensitivity to reward and punishment, and significantly lower on emotion regulation, compared to those in the non-binge group. Hierarchical regression models explained 25% of variance in exercise dependence symptoms in non-binge-eaters and 43% in severe binge eaters. Findings are discussed in terms of the distinction between primary and secondary exercise dependence, the role of alexithymia, study limitations including data collection during the COVID-19 pandemic in Australia, and suggestions for future research.

## Introduction

Regular exercise has well-documented psychological and health benefits (World Health Organization [WHO], 2010), however adverse impacts can also occur such as musculoskeletal injuries or cardiovascular events, especially when exercise is excessive. A small proportion of those who exercise regularly are reported to become psychologically dependent on exercise and exhibit symptoms similar to addictions, such as craving, loss of control, tolerance, withdrawal, continuing the behavior despite adverse impacts such as injuries, and interference with various life domains (Hausenblas & Symons Downs, 2002)*.* Commonly referred to as exercise dependence, alternate terms for this excessive behavior have included exercise addiction, obsessive exercise, compulsive exercise, exercise abuse and others (Egorov & Szabo, 2013).

A review by Sussman et al. (2011) concluded that the prevalence of exercise dependence in the general U.S. population was approximately 3%, however a prevalence of nearly 9% was reported in gym attendees (Manfredi & Gambarini, 2015). Comorbidity has been reported between exercise dependence and eating disorders, thus prompting the distinction between primary and secondary exercise dependence where the latter is defined by its association with eating-related concerns and weight loss motivation (Cook & Hausenblas, 2008; Cunningham et al., 2016; Di Lodovico et al., 2018; Meyer et al., 2011; Van Landeghem et al., 2019). Given that certain trait factors have been consistently linked to other excessive behaviors, including substance and behavioral addictions, the present study examined such traits in relation to symptoms of exercise dependence while attempting to differentiate between primary and secondary types.

Like substance addictions (e.g., Lyvers et al., 2014; Thorberg et al., 2017), exercise dependence is reportedly associated with negative psychological symptoms such as depression and anxiety as well as disruptions to social life and personal relationships, in this case due to an overriding commitment to exercise regimens (Back et al., 2019; Costa et al., 2013; Grandi et al., 2011; Li et al., 2015; Meyer et al., 2011; Weinstein et al., 2015). Exercise dependence entails an elevated risk of musculoskeletal injuries caused by excessive exercise, and an insistence on continuing to exercise despite such injuries, which can prevent proper healing (Freimuth et al., 2011; Lichtenstein et al., 2014). Given such negative outcomes, why would someone become addicted to exercise?

### Potential roles of trait factors: reward sensitivity and alexithymia

Like addictive substances, exercise can elevate mood, in this case both through direct effects of inducing positive mood and reducing stress as well as indirectly via improved appearance- or performance-related self-esteem and self-confidence (Dietrich, 2006; Freimuth et al., 2011). Such benefits may account for the progression and maintenance of exercise regimens, but do not explain why some people who exercise regularly do so excessively whereas most do not, similar to the question of why only a subset of substance users or gamblers become addicted (Egorov & Szabo, 2013). As with other forms of addiction, such considerations raise the question of the role of personality traits as risk factors for exercise dependence. One such trait, sensitivity to reward, is reportedly elevated in excessive substance users and binge eaters (Dawe & Loxton, 2004), presumably because certain drugs, and of course food, have primary reward properties. The rewarding effects of exercise may likewise tend to promote compulsive exercise in those with high reward sensitivity, an idea supported by the findings of a recent study on traits associated with exercise dependence that did not, however, distinguish between primary and secondary types (Lyvers et al., 2021).

Another trait that has been consistently linked to a variety of addictions is alexithymia, a subclinical trait dimension defined by difficulties identifying and describing feelings and an externally oriented thinking style (Bagby et al., 2020). Alexithymia has been linked to risky or problematic alcohol use in both clinical samples of alcohol-dependent clients (Cruise & Becerra, 2018; Thorberg et al., 2009) and in nonclinical samples of young adult alcohol users (Lyvers et al., 2018a, b, 2019, 2020). Alexithymia has also been linked to various other excessive behaviors including excessive use of other drugs (Ghalehban & Besharat, 2011; Lyvers et al., 2013, 2014), binge eating (Marsero et al., 2011; Westwood et al., 2017; Wheeler et al., 2005), pathological gambling (Marchetti et al., 2019; Toneatto et al., 2009), compulsive buying (Rose & Segrist, 2012), internet addiction (Kandri et al., 2014; Lyvers et al., 2016; Mahapatra & Sharma, 2018), and exercise dependence (Lyvers et al., 2021; Van Landeghem et al., 2019).

### Alexithymia and addictive behaviors: mood regulation or interoceptive deficit?

A common explanation as to why alexithymia is associated with excessive behaviors is that alexithymia is characterized by deficits of both emotional self-awareness and emotional self-regulation, promoting use of maladaptive or externalized coping strategies to alleviate distress (Lyvers et al., 2019). Brewer et al. (2016) offered an intriguing alternative possibility that alexithymia is the manifestation of a fundamental deficit of interoceptive awareness, including not only deficient awareness of internal bodily manifestations of emotions but also internal cues of intoxication or overconsumption, perhaps accounting for the associations of alexithymia with excessive alcohol or other drug use or binge eating. Consistent with this idea, brain areas reported to show abnormalities in alexithymia include regions involved in both interoceptive awareness and emotions (Gu et al., 2013; Stevens et al., 2011). Further, alexithymia has been associated with poor perception of heartbeats (Herbert et al., 2011; Murphy et al., 2018), consistent with deficient interoceptive awareness in alexithymia - though the validity of heartbeat counting as an index of interoception has recently been questioned due to identified confounds (Zamariola et al., 2018). In any case, such a deficit, if present in alexithymia, could conceivably account for the association of alexithymia with excessive exercise, as deficient interoception might be expected to promote excessive exercise due to lack of sensitivity to bodily cues of over-exertion or impending injury. The present study thus assessed both emotion regulation and interoceptive awareness in relation to alexithymia and symptoms of exercise dependence, aiming to evaluate the two proposed mechanisms of the association of alexithymia with excessive or addiction-like exercise engagement.

### Alexithymia, reward sensitivity, and binge eating: potential relevance to exercise dependence subtypes

Binge eating disorder (BED) is not only the most common eating disorder, but also the eating disorder most strongly associated with alexithymia (Aloi et al., 2017; Wheeler et al., 2005). BED is defined by frequent overconsumption of food, often when not hungry, accompanied by a sense of loss of control but without the purging or other compensatory behaviors associated with bulimia nervosa (American Psychiatric Association [APA], 2013). In Australia, BED is by far the most common eating disorder, comprising nearly half of all eating disorder diagnoses and reportedly affecting about 6% of the population (National Eating Disorders Collaboration [NEDC], 2017). Other eating disorders tend to be much more prevalent in females than in males, however BED is different in this regard, as BED rates tend to be similar in men and women (Hay et al., 2015). Subclinical binge eating appears to be extremely common in young adults, with rates as high as 30–40% in a U.S. university student sample (Saules et al., 2009). Given that alexithymia has been independently linked to both binge eating and exercise dependence, the question arises as to whether the reported association of alexithymia with excessive exercise may reflect its well-established association with binge eating rather than so-called primary exercise dependence. The same question might arise regarding reward sensitivity, a trait that, like alexithymia, has also been linked to both binge eating and exercise dependence, as noted earlier. The relationships of both alexithymia and reward sensitivity to exercise dependence symptoms might thus vary depending on whether the sample includes binge eaters or not, an issue examined in the present investigation.

### The current study

The present study sought to assess whether symptoms of exercise dependence were differentially associated with alexithymia and reward sensitivity in non-binge versus severe binge eaters, as in the former group such symptoms would presumably be likely to reflect primary exercise dependence, whereas in the latter group such symptoms would be more likely to reflect secondary exercise dependence. A second goal was to evaluate the purported roles of deficient emotion regulation and/or deficient interoception in the association between alexithymia and excessive exercise, based on the theoretical frameworks described earlier. Risky or problematic alcohol use was also assessed for exploratory purposes to see if there was evidence of comorbidity between exercise dependence symptoms and alcohol misuse, given that disordered eating has also shown such comorbidity. The non-binge and severe binge eater groups were subsamples of a larger sample of young Australian adults who were administered validated self-report measures of the traits of interest online.

Based on theoretical considerations stemming from previous research cited earlier on the likely roles of these traits in addiction risk, alexithymia and reward sensitivity were tentatively predicted to show independent positive relationships with exercise dependence symptoms irrespective of whether participants engaged in binge eating or not. This was based on the primary rewarding and mood-altering aspects of exercise noted earlier. A second prediction of the present study was that if alexithymia showed the expected positive association with symptoms of primary and/or secondary exercise dependence symptoms, the relationship would be mediated by deficient emotion regulation and/or deficient interoception based on the respective emotion regulation deficit and interoceptive deficit interpretations of the relationship of alexithymia to addictive behaviors. Hypotheses were tested via comparison of non-binge and severe binge eater groups on the variables of interest, and by separate regression models on exercise dependence symptoms for each group, with the trait measures as predictors.

## Method

### Participants

Approval for the project was granted by the university ethics committee prior to participant recruitment. Participants were recruited through an online survey hosting tool, Qualtrics Panels, and were incentivised for their time by the survey company. Quotas were requested for gender (1:1 male:female) and Australian state of residence proportionate to the population contribution of each state. Inclusion criteria required that participants be 18 to 30 years old. Participants were excluded if they were currently taking medication for a neurological or psychological disorder, or had suffered a traumatic brain injury; this was to reduce potential extraneous sources of variability in responses.

Data were collected from 572 initial participants; after the survey hosting company removed cases with perseverative responses, missing data, or that did not meet criteria for inclusion, the sample consisted of 541 participants. Removal of 13 multivariate outliers identified by Mahalanobis distance (*p* < .001) then yielded a final sample of 528 participants aged 18 to 30 years (*M* = 24.77, *SD* = 3.58), of whom 375 (71%) identified as female and 153 (29%) identified as male. There were 349 (66%) non-students and 179 (34%) current students in the sample. Highest education level achieved was less than high school for 27 (5%) participants, high school for 187 (35%) participants, undergraduate or trade school degree for 244 (46%) participants, and postgraduate degree for 70 (13%) participants.

### Measures

The following questionnaires were completed by all participants in the final sample.

#### Demographics questionnaire

This contained questions requesting information on age, sex, student status, highest education level completed, and (for screening purposes) current use of medication for a psychological or neurological disorder and history of traumatic brain injury.

#### Exercise dependence scale revised (EDS-21; Hausenblas & Symons Downs, 2002)

The EDS-21 is a 21 item self-report questionnaire assessing symptoms of exercise dependence based on DSM-IV (APA, 2000) criteria for substance dependence. Each of the seven criteria indicative of exercise dependence (tolerance, withdrawal, intention effects, lack of control, time, reduction in other activities, and continuance) is assessed by three items. Responses are made on a six-point Likert scale, with options ranging from 1 (never) to 6 (always). Examples of items include “I exercise to avoid feeling irritable” and “I exercise when injured.” Item responses are summed to provide an overall score where higher scores indicate higher levels of exercise dependence symptoms. Cut-off scores are provided for classification of respondents as non-dependent, symptomatic, or dependent. In the current sample, internal consistency reliability of the total EDS-21 was very high (α = .97).

#### Toronto Alexithymia Scale (TAS-20; Bagby et al., 1994a, b)

The TAS-20 contains 20 items assessing alexithymia and has three subscales reflecting three facets of alexithymia: difficultly identifying feelings (DIF; e.g., “I am often confused about what emotion I am feeling”), difficultly describing feelings (DDF; e.g., “It is difficult for me to find the right words for my feelings”), and externally oriented thinking (EOT; e.g., “I prefer to just let things happen rather than to understand why they turned out that way”). Respondents indicate the extent to which they agree or disagree with each statement on a five-point Likert scale ranging from 1 (strongly disagree) to 5 (strongly agree). Five items are reverse scored; summation of responses then yields subscale or total scale scores. Total scores can range from 20 to 100, with higher scores indicating higher levels of alexithymia. Total scores were used in the present study as recommended by the authors of the TAS-20 (see Sekely et al., 2018). The total TAS-20 displayed good internal consistency in the present sample, α = .81.

#### Sensitivity to punishment and sensitivity to reward questionnaire (SPSRQ; Torrubia et al., 2001)

The SPSRQ is a 48-item questionnaire comprised of two scales: sensitivity to reward (SR) and sensitivity to punishment (SP). SR and SP assess the influences of the behavioural approach system (BAS; appetitive motivation) and the behavioural inhibition system (BIS; avoidance motivation) respectively, based on Gray’s (1987) influential theory of brain motivational systems. There are 24 items on the SR scale (even numbered items; e.g., “Do you often do things to be praised?”) and on the SP scale (odd numbered items; e.g., “Are you often afraid of new or unexpected situations?”). Participants respond either Yes (1) or No (0). Affirmative responses are summed to obtain total scores on SR and SP. Higher scores reflect stronger influences of the corresponding brain motivational systems. In the present sample, SR and SP showed good internal consistency, with α = .86 for SP and α = .82 for SR.

#### Negative Mood Regulation Scale (NMRS; Catanzaro & Mearns, 1990)

The NMRS is a 30-item questionnaire designed to measure generalized expectancies to reduce emotional distress by one’s own efforts (e.g., “I’ll feel okay if I think about more pleasant times”). Item responses are anchored on a five-point Likert scale ranging from 1 (strongly disagree) to 5 (strongly agree). Once the 13 negatively worded items are reverse scored, total scores are calculated by summation. Higher scores indicate greater ability to self-regulate emotions to reduce negative moods. According to the authors, the NMRS showed discriminant validity from social desirability, impulsivity, and depression. The NMRS had high internal consistency in the present sample, α = .92.

#### Multidimensional Assessment of Interoceptive Awareness revised (MAIA-2; Mehling et al., 2018)

The MAIA-2 is a 37-item questionnaire assessing eight dimensions of interoception via eight subscales, acknowledging the multidimensional nature of interoception (Suksasilp & Garfinkel, 2022). The Noticing subscale assesses awareness of bodily sensations whether uncomfortable, neutral, or comfortable (e.g., “I notice when I am uncomfortable in my body”). Not-Distracting assesses the extent to which an individual cannot ignore sensations of discomfort or pain (e.g., “I distract myself from sensations of discomfort,” reverse scored). Not-Worrying assess the tendency to not worry about pain or discomfort (e.g., “I can stay calm and not worry when I have feelings of discomfort or pain”). Attention Regulation assesses the ability to maintain and control attention towards bodily sensations (e.g., “I can return awareness to my body if I am distracted”). Emotional Awareness assesses the emotion-body connection (e.g., “I notice how my body changes when I’m angry”). Self-Regulation assesses the ability to pay attention to body sensations to regulate distress (e.g., “I can use my breath to reduce tension”). Body Listening assesses gaining insight from the body by actively listening to the body (e.g., “Listen to my body to inform me about what to do”). Trusting the Body assesses the body’s signals as reliable (e.g., “I trust my body sensations”). Respondents indicate how often the statements apply to them in daily life, using a Likert scale anchored at the extremes with 0 (never) and 5 (always). Nine items are reverse scored. Subscale scores are calculated by summing responses and dividing by the number of items in each subscale. An overall score can be calculated by summing and averaging all items. Higher scores indicate higher levels of interoceptive awareness. In the present sample, the total MAIA-2 displayed good internal consistency, α = .85.

#### Binge-Eating Scale (BES; Gormally et al., 1982)

The BES was used in the present study to classify subsets of the overall sample as non-binge eaters or severe binge eaters. There are 16 items assessing binge eating behaviors and associated cognitions. Items referring to binge eating behaviors have four response options describing non-binge eating (e.g., “I don’t have any difficulty eating slowly in the proper manner”), mild bingeing (e.g., “Although I seem to ‘gobble down’ foods, I don’t end up feeling stuffed because of eating too much”), moderate bingeing (e.g., “At times, I tend to eat quickly and then, I feel uncomfortably full afterwards”), and severe bingeing (e.g., “I have the habit of bolting down my food, without really chewing it”). Other items refer to cognitions and concerns regarding eating, with four response options for all but two items, which have three; response options describe lack of concern about eating (e.g., “I don’t think much about trying to control unwanted eating urges”), mild concern (e.g., “At least some of the time, I feel my thoughts are pre-occupied with trying to control my eating urges”), moderate concern (e.g., “I feel that frequently I spend much time thinking about how much I ate or about trying not to eat anymore”), and severe concern (e.g., “It seems to me that most of my waking hours are preoccupied by thoughts about eating or not eating. I feel like I’m constantly struggling not to eat”). Responses are scored from 0 to 3 (or 0 to 2 for the two items with three response options) and are summed to yield a total score, where higher scores indicate greater severity of binge eating. Total scores can range from 0 to 46, with scores less than 17 indicating little or no binge eating or eating-related concerns, and scores above 27 indicating severe or problematic binge eating (Gormally et al., 1982). In the present sample, the BES displayed high internal consistency, α = .93.

#### Alcohol Use Disorders Identification Test (AUDIT; Babor et al., 2001)

The AUDIT is a widely used screening tool for risky or harmful alcohol use. Items assess frequency of alcohol consumption (3 items), signs of alcohol dependence (3 items), and alcohol-related problems (4 items). Items 1 to 8 are scored on a five-point Likert scale from 0 to 4, with different anchors depending on the question (e.g., “How often during the last year have you failed to do what was normally expected of you because of drinking?” with options of never, less than monthly, monthly, weekly, and daily or almost daily). Items 9 and 10 use a three-point Likert scale with options of 0 (No), 2 (Yes, but not during the last year) and 4 (Yes, during the last year). Each item score ranges from 0 to 4, thus total scores can range from 0 to 40. Scores of 8 or more indicate hazardous drinking, and scores of 16 or more indicate harmful drinking (Saunders et al., 1993). The AUDIT showed high internal consistency in the present sample (α = .88).

### Procedure

Participants accessed an online link to the questionnaire battery. Upon clicking the link, participants were presented with an explanatory statement outlining the purpose of the study as an investigation of personality, body awareness, and health habits such as eating and exercise. They were assured of the anonymity of all responses and their right to withdraw their participation at any time without penalty. Data storage procedures were described, and contact details of the researchers were provided as well as a distress hotline (Lifeline) should they experience distress as a result of their participation. There was a brief disclaimer stating that some of the questions were of a sensitive nature, followed by a question asking if the participant agreed to participate. Those who did not click Yes were immediately released from participation. Those who agreed to participate were presented with the demographic questionnaire first, which included questions assessing whether the participant met inclusion criteria; if their responses indicated they did not, they were automatically exited from the survey and thanked for their time. For those who met inclusion criteria, the demographics questionnaire was followed by the other measures in uniquely randomized orders per participant. Participants had to answer every item per page before they could proceed to the next page. Typical completion time was about 30 minutes, after which participants were thanked for their time. Based on the established BES cut-off scores (Gormally et al., 1982) described earlier, participants were categorized as non-binge, mild to moderate binge, or severe binge eaters, with the first and last comprising the groups of interest for comparison purposes.

### Design

The present study used a cross-sectional correlational design and group comparison. Preliminary analyses included multivariate analysis of covariance (MANCOVA) to compare non-binge to severe binge eater groups on the variables of interest while controlling for sex as the covariate. Pearson correlations were conducted separately for each group to assess associations among continuous measures in each group. Hypotheses were then tested by separate hierarchical linear regressions for each group, controlling for demographic covariates age, sex, highest completed education level, and student status at step 1, the SPSRQ indices of BAS influence (SR) and BIS influence (SP) at step 2, TAS-20 alexithymia at step 3, and both NMRS emotion regulation and overall MAIA-2 interoception at step 4. If the final step suggested mediation of the relationship of alexithymia to exercise dependence symptoms by deficient emotion regulation and/or deficient interoception, then bootstrapped mediation tests were to be conducted separately for each group to evaluate the two competing interpretations of the link between alexithymia and excessive behavior described earlier.

## Results

IBM Statistical Package for Social Sciences (SPSS) version 28 was used for all analyses. For purposes of analysis, demographic variables of sex and student status were dummy coded as binary variables, whereas education was coded 1–4 into the previously described four categories denoting years of education. Skewness and kurtosis were within ± 1 for all continuous variables, indicating normal or near normal distributions. In the overall sample, 290 participants (54%) scored as asymptomatic on the EDS-21, indicating no exercise dependence, whereas 232 participants (44%) scored as symptomatic or at potential risk of exercise dependence, and 10 participants (2%) scored as exercise dependent; the latter percentage was slightly below the 3% population prevalence of exercise dependence previously estimated by Sussman et al. (2011) in a U.S. sample. EDS-21 exercise dependence symptoms were significantly positively correlated with BES binge eating scores in the overall sample, *r*(528) = .22, *p* < .001. Based on BES binge eating scores and using the established cut-offs described earlier (Gormally et al., 1982), 59 males and 169 females were classed as non-binge eaters, 63 males and 115 females as mild to moderate binge eaters, and 31 males and 95 females as severe binge eaters; the proportion of females in the latter group (75%) was nonsignificantly higher than in the non-binge (74%) or mild-moderate binge (65%) groups, χ^2^(2) = 5.81, *p* = .06. Separate correlation and regression analyses were conducted for participants whose BES scores indicated minimal or no binge eating or eating-related concerns (*n* = 228) and for those whose BES scores indicated a severe level of binge eating and eating-related concerns (*n* = 126). These planned analyses necessarily excluded mild to moderate binge eaters.

### Characterization of non-binge versus severe binge eater groups

MANCOVA was used to compare non-binge to severe binge groups on the variables of interest while controlling for sex as the covariate. The omnibus F was significant according to Pillai’s Trace, *F*(7, 341) = 21.00, *p* < .001, partial eta squared = .31, observed power = 1. Between subjects effects were significant for all dependent variables (all *p* < .001) except interoception (*p* = .57). Severe binge eaters scored significantly higher on the EDS-21 index of exercise dependence symptoms (*M* = 61.19, *SD* = 26.00) than non-binge eaters (*M* = 51.14, *SD* = 22.69). Severe binge eaters scored significantly higher on the TAS-20 measure of alexithymia (*M* = 64.30, *SD* = 9.24) than non-binge eaters (*M* = 54.86, *SD* = 10.98). Severe binge eaters scored significantly higher on the SPSRQ indices of sensitivity to reward (*M* = 12.39, *SD* = 5.63) and sensitivity to punishment (*M* = 17.13, *SD* = 4.80) than non-binge eaters (*M* = 9.17, *SD* = 4.58; *M* = 14.14, *SD* = 6.01, respectively). Severe binge eaters scored significantly lower on the NMRS index of emotion regulation (*M* = 88.24, *SD* = 11.04) than non-binge eaters (*M* = 101.83, *SD* = 16.03). Finally, severe binge eaters scored significantly higher on the AUDIT index of risky or problematic alcohol use (*M* = 9.96, *SD* = 9.10) than non-binge eaters (*M* = 4.30, *SD* = 5.10). There was no difference on the overall MAIA-2 index of interoceptive awareness between severe binge eaters (*M* = 2.70, *SD* = .67) and non-binge eaters (*M* = 2.74, *SD* = .73).

### Exercise dependence symptoms in non-binge eaters

Means, standard deviations and Pearson correlations among variables are presented in Table 1 for non-binge eaters. As can be seen in the table, the EDS-21 index of exercise dependence symptoms was significantly positively correlated with the TAS-20 index of alexithymia, the overall MAIA-2 index of interoception, the AUDIT index of risky or problematic alcohol use, and the SPSRQ-SR index of reward sensitivity. Alexithymia was also significantly positively correlated with risky or problematic drinking, reward sensitivity and punishment sensitivity, and was significantly negatively correlated with the NMRS index of emotion regulation as expected, but contrary to expectations alexithymia was uncorrelated with interoception.

**Table 1:** Correlations between exercise dependence symptoms and the other variables in non-binge eaters (*n* = 228)

Variable	*1*	*2*	*3*	*4*	*5*	*6*	*7*
1. Exercise Dep	–						
2. Alexithymia	.13*	–					
3. Emotion Reg	.04	−.47***	–				
4. Interoception	.33***	−.10	.20**	–			
5. Sens Reward	.31***	.17**	−.03	.29***	–		
6. Sens Punish	−.07	.45***	−.43***	.03	.26***	–	
7. Alcohol Risk	.35***	.25**	−.12	.09	.40***	.05	–

*Exercise Dep* Exercise Dependence Symptoms, *Emotion Reg* Emotion Regulation, *Sens Reward* Sensitivity to Reward, *Sens Punish* Sensitivity to Punishment. *p < .05. **p < .01. ***p < .001

When MAIA-2 subscale scores were assessed in place of the overall score in relation to EDS-21 and TAS-20 scores, exercise dependence symptoms were significantly positively correlated with all MAIA-2 subscales except Not Distracting, with correlations ranging from *r* = .21 to *r* = .37, all *p* < .01. Alexithymia showed a significant positive correlation with Not Distracting (*r* = .23, *p* < .001) and significant negative correlations with Attention Regulation (*r* = −.26, *p* < .001) and Trusting the Body (*r* = −.25, *p* < .001).

Hierarchical regression was conducted on exercise dependence symptoms in non-binge eaters, with demographic covariates age, sex, highest completed education level, and student status at step 1, the SPSRQ indices of BAS influence (SR) and BIS influence (SP) at step 2, TAS-20 alexithymia at step 3, and both NMRS emotion regulation and overall MAIA-2 interoception at step 4. Step 1 was not significant, *R*^*2*^ = .02, *F*(4, 221) = 1.25, *p* = .29. Step 2 was significant, *R*^*2*^ = .14, *Fchange*(2, 219) = 14,23, *p* < .001, accounting for 11% of additional variance; only SR was significant and was a positive predictor (see Table 2). Step 3 was also significant, *R*^*2*^ = .17, *Fchange*(1, 218) = 9.52, *p* = .002, with alexithymia accounting for 3.6% of additional variance; SR remained significant, and SP became a significant negative predictor. The final step was again significant, *R*^*2*^ = .25, *Fchange*(2, 216) = 10.96, *p* < .001, explaining 7.6% of additional variance; interoception was significant as a positive predictor but emotion regulation was not significant, with alexithymia, SR and SP remaining significant, and age becoming a positive predictor. The final model accounted for 25% of variance in exercise dependence symptoms in non-binge eaters, *F*(9, 216) = 7.88, *p* < .001. Regression coefficients at each step are displayed in Table 2.

**Table 2:** Hierarchical regression on exercise dependence symptoms in non-binge eaters (*n* = 228)

	Step 1			Step 2			Step 3			Step 4		
	*B*	*SE B*	β	*B*	*SE B*	β	*B*	*SE B*	β	*B*	*SE B*	β
Constant	36.62	15.03		23.34	14.84		−1.83	16.69		−23.86	20.48	
Age	.56	.51	.09	.77	.48	.12	.87	.48	.13	.93	.46	.14*
Sex	−3.95	3.46	−.08	−.52	3.37	−.01	−1.10	3.31	−.02	−2.33	3.18	−.05
Education	−.26	2.33	−.01	−1.19	2.21	−.04	−.29	2.18	−.01	.21	2.10	.01
Student	6,16	3.46	.13	3.60	3.31	.07	4.98	3.28	.10	4.65	3.15	.10
SensRew				1.77	.34	.35***	1.67	.33	.34***	1.21	.33	.24***
SensPun				−.58	.25	−.15*	−.91	.27	−.24***	−.95	.27	−.25***
Alexithymia							.46	.15	.22**	.55	.15	.26***
EmoReg										−.04	.10	−.03
Interocept										9.21	1.98	.30***

*B* unstandardized coefficient, *SE B* standard error of *B, β* standardized coefficient, *SensRew* Sensitivity to Reward, *SensPun* Sensitivity to Punishment, *EmoReg* Emotion Regulation, *Interocept* Interoception. *p < .05. **p < .01. ***p < .001

### Exercise dependence symptoms in severe binge eaters

Means, standard deviations and Pearson correlations among variables are presented in Table 3 for severe binge eaters. As can be seen in the table, the EDS-21 index of exercise dependence symptoms was uncorrelated with the TAS-20 index of alexithymia in severe binge eaters, but was significantly positively correlated with the overall MAIA-2 index of interoception, the AUDIT index of risky alcohol use, and the SPSRQ-SR index of reward sensitivity, and was negatively correlated with the SPSRQ-SP index of punishment sensitivity. Alexithymia was significantly positively correlated with punishment sensitivity, and significantly negatively correlated with the NMRS index of emotion regulation as expected, but was not significantly correlated with interoception or alcohol-related risk; the latter was significantly positively correlated with reward sensitivity.

**Table 3:** Correlations between exercise dependence symptoms and the other variables in severe binge eaters (*n* = 126)

Variable	*1*	*2*	*3*	*4*	*5*	*6*	*7*
1. Exercise Dep	–						
2. Alexithymia	−.07	–					
3. Emotion Reg	.12	−.45***	–				
4. Interoception	.35***	.01	.20*	–			
5. Sens Reward	.36***	.16	.07	.31***	–		
6. Sens Punish	−.27**	.36***	−.25**	.02	.37***	–	
7. Alcohol Risk	.49***	.11	−.05	.14	.35***	−.03	–

*Exercise Dep* Exercise Dependence Symptoms, *Emotion Reg* Emotion Regulation, *Sens Reward* Sensitivity to Reward, *Sens Punish* Sensitivity to Punishment. *p < .05. **p < .01. ***p < .001

When MAIA-2 subscale scores were assessed in place of the overall score in relation to EDS-21 and TAS-20 scores, exercise dependence symptoms were significantly positively correlated with four of the eight MAIA-2 subscales: Attention Regulation (*r* = .37, *p* < .001), Self-Regulation (*r* = .35, *p* < .001), Body Listening (*r* = .51, *p* < .001), and Trusting the Body (*r* = .41, *p* < .001). Alexithymia showed only a significant positive correlation with Noticing (*r* = .30, *p* < .001).

Hierarchical regression was conducted on exercise dependence symptoms in severe binge eaters, with demographic covariates age, sex, highest completed education level, and student status at step 1, the SPSRQ indices of BAS influence (SR) and BIS influence (SP) at step 2, TAS-20 alexithymia at step 3, and both NMRS emotion regulation and overall MAIA-2 interoception at step 4. Step 1 was significant, *R*^*2*^ = .22, *F*(4, 119) = 8.23, *p* < .001; sex was the only significant predictor. Independent t-test showed that exercise dependence symptoms were significantly higher in men with severe binge eating (*M* = 80.42, *SD* = 17.39, *n* = 31) than in women with severe binge eating (*M* = 54.78, *SD* = 25.28, *n* = 93), *t*(122) = 5.24, *p* < .001. Step 2 was also significant, *R*^*2*^ = .39, *Fchange*(2, 117) = 16.66, *p* < .001, accounting for 17.4% of additional variance; SR was a significant positive predictor and SP a significant negative predictor, with sex remaining significant (see Table 4 ). Step 3 was not significant, *R*^*2*^ = .39, *Fchange*(1, 116) < 1; alexithymia accounted for 0% of additional variance, with SR, SP, and sex remaining significant. The final step was significant, *R*^*2*^ = .43, *Fchange*(2, 114) = 3.51, *p* = .03, explaining 4% of additional variance; interoception was significant as a positive predictor but emotion regulation was not significant, with sex, SR and SP remaining significant. The final model accounted for 43% of variance in exercise dependence symptoms in severe binge eaters, *F*(9, 114) = 9.45, *p* < .001. Regression coefficients at each step are displayed in Table 4.

**Table 4:** Hierarchical regression on exercise dependence symptoms in severe binge eaters (*n* = 126)

	Step 1			Step 2			Step 3			Step 4		
	*B*	*SE B*	β	*B*	*SE B*	β	*B*	*SE B*	β	*B*	*SE B*	β
Constant	92.62	18.94		76.59	17.96		64.61	23.30		77.21	31.28	
Age	.00	.64	.00	.56	.58	.08	.60	.58	.08	.33	.59	.05
Sex	−27.17	4.94	−.45***	−15.51	4.81	−.26**	−15.56	4.82	−.26**	−14.88	4.76	−.25**
Education	1.36	2.66	.04	1.98	2.44	.06	2.27	2.47	.07	2.71	2.44	.09
Student	9.49	4.57	.18*	2.80	4.24	.05	3.15	4.27	.06	1.90	4.23	.04
SensRew				2.11	.40	.46***	2.11	.40	.46***	1.92	.42	.42***
SensPun				−2.15	.45	-.41***	−2.24	.46	−.43***	−2.19	.47	−.42***
Alexithymia							.18	.22	.06	.08	.23	.03
EmoReg										−.19	.20	−.08
Interocept										6.78	2.92	.18*

*B* unstandardized coefficient, *SE B* standard error of *B*, *β* standardized coefficient, *SensRew* Sensitivity to Reward, *SensPun* Sensitivity to Punishment, *EmoReg* Emotion Regulation, *Interocept* Interoception. *p < .05. **p < .01. ***p < .001

Anticipated mediation tests in both groups to assess the potential roles of deficient emotion regulation versus deficient interoception in the relationship of alexithymia with exercise dependence symptoms were not conducted because (1) the measure of emotion regulation did not predict exercise dependence symptoms in the regressions for either group, and (2) the measure of interoception was a positive predictor of exercise dependence symptoms rather than a negative predictor, contrary to expectations based on the idea that deficient interoception might promote excessive exercise due to lack of sensitivity to bodily cues of over-exertion or impending injury. Further, alexithymia was unrelated to exercise dependence symptoms in the severe binge eating group.

## Discussion

The present study yielded some findings that were expected and some that were unexpected. The SPSRQ-SR index of reward sensitivity showed a positive relationship with exercise dependence symptoms in both non-binge and severe binge eater groups alike, consistent with previous work that did not distinguish between primary and secondary exercise dependence (Lyvers et al., 2021), and presumably reflecting the rewarding psychological and health benefits of exercise (Back et al., 2019; Dietrich, 2006; Freimuth et al., 2011; WHO, 2010). By contrast, the SPSRQ-SP index of punishment sensitivity was negatively related to exercise dependence symptoms in both groups. This result could conceivably be explained by an aversion to extreme exertion or risk of pain in those who scored high on SP, which has been associated with high neuroticism (Lee-Winn et al., 2016) and high alexithymia (Lyvers et al., 2012) in previous work; the positive association of SP with alexithymia was replicated in the present study.

Alexithymia as measured by the TAS-20, which was reported to show a positive association with exercise dependence symptoms in previous work (Lyvers et al., 2021), only showed such an association in non-binge eaters in the present study, presumably reflecting a role of alexithymia in primary but not secondary exercise dependence. In severe binge eaters, alexithymia was unrelated to exercise dependence symptoms, which might be explained by the high SP observed in the severe binge eater group (their mean SPSRQ-SP score was nearly double that in the non-binge eater group) given the negative association of SP with exercise dependence symptoms and the positive association of alexithymia with SP. In other words, those with high levels of SP may tend to regard intense exercise as aversive or potentially harmful, and severe binge eaters showed considerably higher scores on both the SPSRQ-SP index of punishment sensitivity and the TAS-20 measure of alexithymia compared to the non-binge eater group. Thus given the high SP scores in severe binge eaters, and the positive association of SP with alexithymia, high SP could have offset any potential contribution of alexithymia to excessive exercise in that group despite alexithymia being positively associated with exercise dependence symptoms in non-binge eaters, who showed much lower SP scores. Another unexpected difference between the groups was that in the regression models, sex was a significant predictor of exercise dependence symptoms in the severe binge eater group but not in the non-binge eater group, indicating a positive relationship of male sex with such symptoms in severe binge eaters only. Put simply, male severe binge eaters appeared more likely to engage in excessive exercise than female severe binge eaters, an association possibly accounted for by male excessive exercisers engaging in binge eating to “bulk up” (e.g., Fetters & Boly, 2021) and/or allowing themselves to over-indulge in food due to a belief that their caloric expenditure would offset any unwanted weight gain (Stapleton et al., 2014).

An unexpected finding of the present study was that there was no evidence consistent with either the emotion regulation deficit (Lyvers et al., 2019) or interoceptive awareness deficit (Brewer et al., 2016) hypotheses of the relationship between alexithymia and excessive behaviors, in this case excessive exercise. The NMRS measure of emotion regulation was unrelated to exercise dependence symptoms in both non-binge and severe binge eater groups, and the overall MAIA-2 index of interoception showed a positive relationship with exercise dependence symptoms in both groups instead of the predicted negative relationship, which was based on the notion that poor interoceptive awareness of over-exertion or impending injury might promote excessive exercise. Results thus suggest that at least according to these measures, neither the presumed emotion regulation effects of exercise, nor deficient interoceptive awareness of bodily signs of over-exertion or impending injury, showed evidence of a link to exercise dependence symptoms in the present samples, and thus may not explain the apparent link of alexithymia to excessive exercise in non-binge eaters in the present study. An alternative possibility is that alexithymia is associated with a tendency to impulsive action, to “do” rather than “think” (Shishido et al., 2013), and that in those who are not severe binge eaters this inclination to “do” can lead to excessive exercise (or “overdo”) in a subset of alexithymic exercisers.

The positive relationship of interoceptive awareness with exercise dependence symptoms in both groups in the present study was not anticipated. However, the MAIA-2 measure of interoception has been reported to yield higher scores in experienced yoga practitioners than in inexperienced ones (Mehling et al., 2012), which would seem to parallel the present results. Perhaps not only yoga, but other forms of exercise as well, may tend to improve bodily self-awareness when done regularly. Another interesting finding of the present study was the substantial positive correlation of the EDS-21 index of exercise dependence symptoms with the AUDIT index of risky or problematic drinking in both non-binge and severe binge eater groups. Although the severe binge eater group scored significantly higher on the AUDIT than the non-binge eater group, the AUDIT was similarly positively correlated with exercise dependence symptoms in both groups. By contrast, a previous study of German fitness center attendees (Müller et al., 2015), using the EDS-21 and AUDIT as in the present study, found no relationship between symptoms of exercise dependence and risky or problematic drinking. The association found in the present sample could reflect a general impulsive tendency toward excessive behaviors (Kane et al., 2004; Sussman et al., 2011), a tendency to use alcohol to alleviate sympathetic arousal or other negative bodily sensations following intense exercise, cultural factors in Australia that promote heavy drinking in heavy exercisers, and/or any number of other unexamined possibilities and merits further investigation.

### Limitations

The present study had some important limitations to consider. The regression models in the present study explained 25% of variance in exercise dependence symptoms in non-binge-eaters and 43% in severe binge eaters, with other sources of variance unaccounted for. Potentially relevant variables not assessed in the present study include rash impulsiveness (Dawe & Loxton, 2004; Shishido et al., 2013) as well as BMI and body satisfaction; future research on primary and secondary exercise dependence should incorporate these variables in more complex models. Further, although the study aimed to recruit a sex-balanced sample, this was not achieved; 71% of the overall sample identified as female, though the sex imbalance was addressed by covariate analysis in both the MANCOVA and in the regressions.

A cross-sectional design using self-report indices of the constructs of interest can show associations among self-report variables, and thus suggest possible directions for more ambitious research, but cannot establish causation and is limited by the degree to which the self-report measures are valid indices of the relevant constructs. Although the present findings did not support the emotion regulation deficit (Lyvers et al., 2019) nor interoceptive deficit (Brewer et al., 2016) interpretations of the relationship between alexithymia and primary exercise dependence, and did not support a potential role of alexithymia in secondary exercise dependence at all, the use of self-report indices of alexithymia and interoception can be questioned. Established scales of the relevant constructs were used, however self-report measures assume that respondents have accurate knowledge of themselves, and such measures can be subject to desirability biases and shared method variance. People who have poor or biased understanding of themselves may not be capable of giving accurate responses on self-report measures of emotional or interoceptive self-awareness. Research that has supported deficient interoception in alexithymia, as measured by the TAS-20, used perception of heartbeats as an objective index of interoceptive awareness (Herbert et al., 2011; Murphy et al., 2018); however recent evidence indicates that the heartbeat counting task may not be a valid index of interoception due to multiple confounds (Zamariola et al., 2018). The present study used the MAIA-2, a multidimensional self-report index of interoceptive awareness. Although the MAIA-2 was reported by its authors to distinguish in expected ways between experienced and inexperienced mind-body therapy and yoga practitioners (Mehling et al., 2012), the MAIA-2 should be evaluated in relation to objective measures of interoception (other than the problematic heartbeat counting task) to further assess its construct validity. As for the TAS-20, it is the most widely used index of alexithymia, in part due to its sound psychometric structure and convergence with clinician ratings (Bagby et al., 2020; Ogrodniczuk et al., 2018; Thorberg et al., 2010). Nevertheless, Ogrodniczuk et al. suggested that both self-report and clinician ratings should be used together to yield the most accurate assessment of alexithymia levels.

The present samples were characterized by unusually high TAS-20 alexithymia scores compared to general Australian population estimates (McGillivray et al., 2016). Similarly elevated mean alexithymia scores have previously been reported in samples recruited online, and were attributed to highly alexithymic individuals tending to spend more time on the internet compared to those with low or no alexithymia (Lyvers et al., 2021) given the association of alexithymia with excessive internet use and internet addiction (Mahapatra & Sharma, 2018). However the present high alexithymia scores could be related to another factor: the data were collected during the COVID-19 pandemic when many regions of Australia were subjected to lockdowns, social distancing, changes to employment, and travel restrictions, with associated negative impacts on mental health – especially in young adults (Newby et al., 2020; Rossell et al., 2021). Although much evidence suggests that alexithymia is a stable personality trait with a likely early developmental onset (Hiirola et al., 2017; Lyvers et al., 2019; Salminen et al., 2006; Tolmunen et al., 2011), alexithymia can in some cases be an acute response to depression or stress (Messina et al., 2014), hence the elevated levels in the current sample could in part reflect responses to the adverse pandemic circumstances and employment-related or social stress during the data collection period. In any case the unexpectedly high mean level of self-reported alexithymia in the current sample may limit the generalizability of the findings.

Finally, the current findings were from nonclinical samples and cannot be assumed to generalize to clinical samples. The BES used in the present study to distinguish non-binge from severe binge eaters is a screening tool that is not diagnostic of BED, and only measures one type of disordered eating; in addition, exercise dependence as assessed by the EDS-21 is not a recognized diagnostic category at present. On the other hand, current conceptualizations of addictive behaviors regard them as distributed continuously in the population, with diagnosed disorders at the extreme end of such distributions (APA, 2013; SAMHSA, 2016); hence the present findings may nevertheless be at least somewhat relevant to the excessive exercise behaviors assessed.

## Conclusions

The findings of the present study suggest that although reward sensitivity is positively associated with exercise dependence symptoms in both non-binge and severe binge eaters, alexithymia may be associated with symptoms of primary but not secondary exercise dependence. The potential reasons for this apparent difference are unclear. Perhaps severe binge eaters with alexithymia have learned to rely on food for affect regulation, with exercise used only to offset unwanted weight gain. Further, the results did not provide support for the emotion regulation deficit or interoceptive deficit interpretations of the apparent link between alexithymia and primary exercise dependence symptoms. Future work could examine the potential roles of other trait factors, such as rash impulsiveness (Dawe & Loxton, 2004; Shishido et al., 2013), in the relationship between alexithymia and primary exercise dependence, as well as assessing other potentially relevant factors such as BMI and body satisfaction in relation to primary and/or secondary exercise dependence. Overall, the findings of the present study suggest that further investigation of the potential roles of personality traits and other factors in primary and secondary exercise dependence is warranted.

## Data Availability

The data for this study are available from the corresponding author on request.
